# High complication rate in Crohn’s disease surgery following percutaneous drainage of intra-abdominal abscess: a multicentre study

**DOI:** 10.1007/s00384-022-04183-x

**Published:** 2022-05-23

**Authors:** Valerio Celentano, Mariano Cesare Giglio, Gianluca Pellino, Matteo Rottoli, Gianluca Sampietro, Antonino Spinelli, Francesco Selvaggi, on behalf of the Italian Society of Colorectal Surgery SICCR, Valerio Celentano, Gianluca Pellino, Matteo Rottoli, Gilberto Poggioli, Giuseppe Sica, Mariano Cesare Giglio, Michela Campanelli, Claudio Coco, Gianluca Rizzo, Francesco Sionne, Francesco Colombo, Gianluca Sampietro, Giulia Lamperti, Diego Foschi, Ferdinando Ficari, Ludovica Vacca, Marta Cricchio, Francesco Giudici, Lucio Selvaggi, Guido Sciaudone, Roberto Peltrini, Andrea Manfreda, Luigi Bucci, Raffaele Galleano, Omar Ghazouani, Luigi Zorcolo, Simona Deidda, Angelo Restivo, Andrea Braini, Francesca Di Candido, Matteo Sacchi, Michele Carvello, Stefania Martorana, Giovanni Bordignon, Imerio Angriman, Angela Variola, Mirko Di Ruscio, Giuliano Barugola, Andrea Geccherle, Francesca Paola Tropeano, Gaetano Luglio, Marta Tanzanu, Diego Sasia, Marco Migliore, Maria Carmela Giuffrida, Enrico Marrano, Gianluigi Moretto, Harmony Impellizzeri, Gaetano Gallo, Giuseppina Vescio, Giuseppe Sammarco, Giovanni Terrosu, Giacomo Calini, Andrea Bondurri, Anna Maffioli MD, Gloria Zaffaroni, Andrea Resegotti, Massimiliano Mistrangelo, Marco Ettore Allaix, Fiorenzo Botti, Matteo Prati, Luigi Boni, Serena Perotti, Michela Mineccia, Antonio Giuliani, Lucia Romano, Giorgio Maria Paolo Graziano, Luigi Pugliese, Andrea Pietrabissa, Gian Gaetano Delaini, Antonino Spinelli, Francesco Selvaggi

**Affiliations:** 1 Chelsea and Westminster Hospital NHS Foundation Trust, London, UK; 2 University of Portsmouth, Portsmouth, UK; 3 Department of Surgery and Cancer, Imperial College, London, UK; 4 Department of Clinical Medical and Surgery, Federico II University, Naples, Italy; 5 Department of Advanced Medical and Surgical Science, Universita’ Degli Studi Della Campania Luigi Vanvitelli, Naples, Italy; 6 Surgery of the Alimentary Tract, IRCCS Azienda Ospedaliero Universitaria di Bologna, Bologna, Italy; 7 Alma Mater, Studiorum University of Bologna, Bologna, Italy; 8 Division of General and HPB Surgery, ASST Rhodense, Rho Memorial Hospital, Milan, Italy; 9 Department of Biomedical Sciences, Humanitas University, Via Rita Levi Montalcini 4, 20090 Pieve Emanuele, Milan, Italy; 10 IRCCS Humanitas Research Hospital, via Manzoni 56, 20089 Rozzano, Milan, Italy

**Keywords:** Crohn’s disease, Inflammatory bowel disease, Intra-abdominal abscess, Colorectal surgery

## Abstract

**Introduction:**

Intra-abdominal abscesses complicating Crohn’s disease (CD) present an additional challenge as their presence can contraindicate immunosuppressive treatment whilst emergency surgery is associated with high stoma rate and complications. Treatment options include a conservative approach, percutaneous drainage, and surgical intervention. The current multicentre study audited the short-term outcomes of patients who underwent preoperative radiological drainage of intra-abdominal abscesses up to 6 weeks prior to surgery for ileocolonic CD.

**Methods:**

This is a retrospective, multicentre, observational study promoted by the Italian Society of Colorectal Surgery (SICCR), including all adults undergoing ileocolic resection for primary or recurrent CD from June 2018 to May 2019. The outcomes of patients who underwent radiological guided drainage prior to ileocolonic resection were compared to the patients who did not require preoperative drainage. Postoperative morbidity within 30 days of surgery was the primary endpoint. Postoperative length of hospital stay (LOS) and anastomotic leak rate were the secondary outcomes.

**Results:**

Amongst a group of 575 included patients who had an ileocolic resection for CD, there were 36 patients (6.2%) who underwent abscess drainage prior to surgery. Postoperative morbidity (44.4%) and anastomotic leak (11.1%) were significantly higher in the group of patients who underwent preoperative drainage.

**Conclusions:**

Patients with Crohn’s disease who require preoperative radiological guided drainage of intra-abdominal abscesses are at increased risk of postoperative morbidity and septic complications following ileocaecal or re-do ileocolic resection.

**Supplementary information:**

The online version contains supplementary material available at 10.1007/s00384-022-04183-x.

## Introduction

Complications of Crohn’s disease (CD) such as obstruction, fistulae, and abscesses represent a common indication for surgical treatment. Intra-abdominal abscesses in patients with CD can be intraperitoneal, retroperitoneal, or intra-mesenteric and typically result from a perforation [1, 2]. Abscesses, or collections, present an additional challenge in the management of active CD as their presence can contraindicate immunosuppressive treatment.

Treatment options in cases of intra-abdominal collections include a conservative approach with percutaneous drainage and antimicrobial therapy or surgical intervention. Nonetheless, the perioperative morbidity associated with surgical resection during the acute stage of sepsis suggests that, when possible, percutaneous drainage should be attempted first [3].

The Italian Society of Colorectal Surgery (SICCR) recently reported the results of a national multicentre study collecting benchmark data on surgical treatment of CD, highlighting significant variations in practice [4]. The current study audited the short-term outcomes of patients who underwent preoperative radiological drainage of intra-abdominal abscesses up to 6 weeks prior to surgery for ileocolonic CD.

## Methods

### Study settings

The SICCR promoted the snapshot study “Current Status of Crohn’s Disease Surgery”, which is a retrospective, multicentre, observational study. A steering committee developed the study protocol following the STROBE checklist [5], and this was reviewed independently by the research board of the SICCR. Ethical approval was obtained from the promoting centres and every participating hospital had a named principal investigator, liaising with the local ethics committee. Obtaining informed consent from the patients was deemed not necessary by the Ethics Committees in view of the retrospective and observational nature of the study. Participating centres were invited directly and by an open call published on the SICCR website and disseminated during a 2-month period via the society newsletter.

### Eligibility criteria

All patients (aged 16 or older), undergoing elective or emergency ileocolic resection for primary or recurrent CD from 1 June 2018 to 31 May 2019, were eligible for participation in the study. Patients undergoing proctocolectomy, proctectomy, or segmental colectomy were excluded. Indication for surgery included limited terminal ileal disease, CD refractory to medical treatment, obstruction, internal fistulae, and abscesses.

### Study objectives

The outcomes of patients who underwent radiological guided drainage for ileocolonic CD complicated by intra-abdominal abscess followed by ileocaecal or ileocolonic resection were extracted from the study population including all patients who underwent surgery for ileocolonic CD within the 12-month study period. Postoperative morbidity within 30 days of surgery was the primary endpoint. Postoperative length of hospital stay (LOS) and anastomotic leak rate were the secondary outcomes.

### Data collection

Collected data included the following: patients’ demographics, Montreal classification, preoperative medical treatment and indication for surgery, American Society of Anaesthesiologists (ASA) grade, operative details, surgical access and conversion rate, length of hospital stay, 30-day postoperative morbidity, readmissions, and reoperations.

Postoperative morbidity was defined as any complication occurring during the hospital stay or within 30 days after surgery, whilst all readmissions were recorded up to 30 days after discharge.

### Statistical analysis

Categorical variables are presented as frequency and percentages and were compared using the chi-square test or Fisher’s exact test, as appropriate. Continuous variables are presented as median (interquartile range) according to their distribution and were compared with the use of the Mann–Whitney *U* test. To identify variables associated with binary outcomes, uni- and multivariable logistic regression analyses were performed. Variables having a *p* value equal to 0.10 or less at the univariable analysis were included in the multivariable model. The odds ratio (ORs) with a 95% confidence interval (CI) was estimated as a measure of association. All reported *p* values were two-tailed, and *p* values of less than 0.05 were statistically significant. Statistical analysis was performed by using R version 3.6.1 (2019, The R Foundation for Statistical Computing).

## Results

A total of 575 patients were included, and 36 patients (6.2%) underwent abscess drainage prior to surgery. Patients’ details are reported in Table 1.

**Table 1:** Patients’ characteristics

			Preoperative drainage		
		All patients (*n* = 575)	No (*n* = 539)	Yes (*n* = 36)	*p*
Age, *median (IQR)*		45.0 (32.0 to 56.0)	45.0 (31.5 to 57.0)	40.5 (32.8 to 51.5)	*0.485*
Gender	M	346 (60.2)	322 (59.7)	24 (66.7)	*0.484*
	F	229 (39.8)	217 (40.3)	12 (33.3)	
BMI, *median (IQR)*		22.0 (19.2 to 24.9)	22.0 (19.5 to 25.0)	20.2 (17.8 to 22.8)	*0.034*
ASA	1	65 (11.5)	64 (12.1)	2 (5.6)	*0.091*
	2	414 (73.4)	390 (73.9)	24 (66.7)	
	3	76 (13.5)	67 (12.7)	9 (25.0)	
	4	8 (1.4)	7 (1.3)	1 (2.8)	
Primary	Yes	365 (63.5)	349 (64.7)	16 (44.4)	*0.019*
	Recurrent	210 (36.5)	190 (35.3)	20 (55.6)	
Montreal A	Missing data	24 (4.2)	23 (4.3)	1 (2.8)	*0.068*
	1	119 (20.7)	105 (19.5)	14 (38.9)	
	2	252 (43.8)	239 (44.3)	13 (36.1)	
	3	180 (31.3)	172 (31.9)	8 (22.2)	
Montreal L	1	239 (41.6)	225 (41.7)	14 (38.9)	*0.034*
	2	25 (4.3)	20 (3.7)	5 (13.9)	
	3	311 (54.1)	293 (54.4)	17 (47.2)	
Montreal B	1	21 (3.7)	18 (3.3)	3 (8.3)	*0.001*
	2	375 (65.2)	362 (67.2)	13 (36.1)	
	3	179 (31.1)	159 (29.5)	20 (55.6)	
Previous surgery	No	281 (49.0)	266 (49.4)	15 (41.7)	*0.394*
	Yes	293 (51.0)	272 (50.6)	21 (58.3)	
Timing of surgery	Elective	488 (85.0)	463 (86.1)	25 (69.4)	*0.027*
	Urgent	78 (13.6)	68 (12.6)	10 (27.8)	
	Emergency	8 (1.4)	7 (1.3)	1 (2.8)	
Associated surgery	No	434 (75.5)	404 (75.0)	30 (83.3)	*0.320*
	Yes	141 (24.5)	135 (25.0)	6 (16.7)	
Preoperative TPN	No	505 (88.1)	478 (89.0)	27 (75.0)	*0.027*
	Yes	68 (11.9)	59 (11.0)	9 (25.0)	
Preoperative therapy with steroids	No	378 (65.7)	354 (65.7)	24 (66.7)	*1.000*
	Yes	197 (34.3)	185 (34.3)	12 (33.3)	
Preoperative therapy with biologics	No	485 (84.5)	459 (85.3)	26 (72.2)	*0.053*
	Yes	89 (15.5)	79 (14.7)	10 (27.8)	
Preoperative therapy with azathioprine	No	505 (87.8)	476 (88.3)	29 (80.6)	*0.185*
	Yes	70 (12.2)	63 (11.7)	7 (19.4)	

### Overall morbidity and anastomotic leak rate

Postoperative morbidity was 44.4% in the group of patients who underwent preoperative abscess drainage, with a wound infection rate of 8.3% and anastomotic leak rate of 11.1%. Amongst the entire study population, 142 patients had postoperative complications (24.6%), with postoperative morbidity and septic complications being significantly higher in the group of patients who underwent preoperative drainage, as shown in Table 2.

**Table 2:** Postoperative outcomes

			Preoperative drainage		
*Outcome*		All patients (*n* = 575)	No (*N* = 539)	Yes (*N* = 36)	*p*
Postoperative morbidity	No	433 (75.3)	413 (76.6)	20 (55.6)	0.008
	Yes	142 (24.7)	126 (23.4)	16 (44.4)	
Wound infection	No	554 (96.3)	521 (96.7)	33 (91.7)	0.138
	Yes	21 (3.7)	18 (3.3)	3 (8.3)	
Intra-abdominal collection	No	552 (96.0)	522 (96.8)	30 (83.3)	0.002
	Yes	23 (4.0)	17 (3.2)	6 (16.7)	
Anastomotic leak	No	557 (96.9)	525 (97.4)	32 (88.9)	0.021
	Yes	18 (3.1)	14 (2.6)	4 (11.1)	
Reoperation	No	543 (94.6)	514 (95.5)	29 (80.6)	0.002
	Yes	31 (5.4)	24 (4.5)	7 (19.4)	
Readmission	No	537 (93.9)	506 (94.4)	31 (86.1)	0.060
	Yes	35 (6.1)	30 (5.6)	5 (13.9)	
LOS, *median (IQR)*		7.0 (6.0 to 9.0)	7.0 (6.0 to 9.0)	10.0 (6.8 to 15.0)	< 0.001

Risk factors for morbidity and anastomotic leak are evaluated in Tables 3 and 4.

**Table 3:** Risk factors for postoperative morbidity

Morbidity		No	Yes	OR (univariable)	OR (multivariable)
Age, *mean (SD)*		44.1 (15.4)	47.3 (15.5)	1.01 (1.00–1.03, *p* = 0.035)	1.01 (0.99–1.03, *p* = 0.180)
Gender	M	257 (74.3)	89 (25.7)	-	-
	F	176 (76.9)	53 (23.1)	0.87 (0.59–1.28, *p* = 0.483)	-
BMI, *mean (SD)*		22.3 (4.1)	21.9 (4.1)	0.97 (0.92–1.02, *p* = 0.282)	-
ASA	1	55 (83.3)	11 (16.7)	-	-
	2	317 (76.6)	97 (23.4)	1.53 (0.80–3.19, *p* = 0.224)	0.97 (0.48–2.12, *p* = 0.945)
	3	47 (61.8)	29 (38.2)	3.09 (1.42–7.07, *p* = 0.006)	1.70 (0.70–4.28, *p* = 0.247)
	4	3 (37.5)	5 (62.5)	8.33 (1.79–45.79, *p* = 0.008)	4.44 (0.71–38.13, *p* = 0.128)
Primary	Yes	290 (79.5)	75 (20.5)	-	-
	Recurrent	143 (68.1)	67 (31.9)	1.81 (1.23–2.66, *p* = 0.003)	1.39 (0.81–2.41, *p* = 0.236)
Montreal A	Missing data	22 (91.7)	2 (8.3)	-	-
	1	89 (74.8)	30 (25.2)	3.71 (1.01–24.00, *p* = 0.088)	3.98 (0.98–27.60, *p* = 0.090)
	2	187 (74.2)	65 (25.8)	3.82 (1.09–24.28, *p* = 0.075)	4.33 (1.10–29.60, *p* = 0.068)
	3	135 (75.0)	45 (25.0)	3.67 (1.02–23.45, *p* = 0.087)	4.08 (0.97–29.09, *p* = 0.091)
Montreal L	1	186 (77.8)	53 (22.2)	-	-
	2	20 (80.0)	5 (20.0)	0.88 (0.28–2.29, *p* = 0.803)	-
	3	227 (73.0)	84 (27.0)	1.30 (0.88–1.93, *p* = 0.194)	-
Montreal B	1	18 (85.7)	3 (14.3)	-	-
	2	297 (79.2)	78 (20.8)	1.58 (0.52–6.85, *p* = 0.475)	2.12 (0.61–10.12, *p* = 0.281)
	3	118 (65.9)	61 (34.1)	3.10 (1.00–13.61, *p* = 0.078)	3.72 (1.06–17.89, *p* = 0.061)
Previous surgery	No	223 (79.4)	58 (20.6)	-	-
	Yes	209 (71.3)	84 (28.7)	1.55 (1.05–2.28, *p* = 0.026)	1.22 (0.70–2.11, *p* = 0.473)
Timing of surgery	Elective	380 (77.9)	108 (22.1)	-	-
	Urgent	46 (59.0)	32 (41.0)	2.45 (1.48–4.02, *p* < 0.001)	1.98 (1.11–3.53, *p* = 0.020)
	Emergency	6 (75.0)	2 (25.0)	1.17 (0.17–5.17, *p* = 0.847)	0.74 (0.05–7.20, *p* = 0.807)
Associated surgery	No	338 (77.9)	96 (22.1)	-	-
	Yes	95 (67.4)	46 (32.6)	1.70 (1.12–2.58, *p* = 0.013)	1.80 (1.13–2.85, *p* = 0.013)
Preoperative drainage	No	413 (76.6)	126 (23.4)	-	-
	Yes	20 (55.6)	16 (44.4)	2.62 (1.30–5.20, *p* = 0.006)	2.28 (1.05–4.88, *p* = 0.034)
Preoperative total parenteral nutrition	No	391 (77.4)	114 (22.6)	-	-
	Yes	41 (60.3)	27 (39.7)	2.26 (1.32–3.82, *p* = 0.003)	1.22 (0.65–2.25, *p* = 0.521)
Preoperative therapy with steroids	No	284 (75.1)	94 (24.9)	-	-
	Yes	149 (75.6)	48 (24.4)	0.97 (0.65–1.45, *p* = 0.895)	-
Preoperative therapy with azathioprine	No	382 (75.6)	123 (24.4)	-	-
	Yes	51 (72.9)	19 (27.1)	1.16 (0.64–2.00, *p* = 0.613)	-
Preoperative therapy with biologics	No	363 (74.8)	122 (25.2)	-	-
	Yes	69 (77.5)	20 (22.5)	0.86 (0.49–1.45, *p* = 0.590)	-

**Table 4:** Risk factors for anastomotic leak

Anastomotic leak		No	Yes	OR (univariable)	OR (multivariable)
Age, mean (SD)		44.7 (15.5)	49.7 (14.6)	1.02 (0.99–1.05, *p* = 0.182)	-
Gender	M	335 (96.8)	11 (3.2)	-	-
	F	222 (96.9)	7 (3.1)	0.96 (0.35–2.48, *p* = 0.934)	-
BMI, mean (SD)		22.2 (4.1)	22.8 (4.3)	1.04 (0.91–1.16, *p* = 0.565)	-
ASA	1	64 (97.0)	2 (3.0)	-	-
	2	403 (97.3)	11 (2.7)	0.87 (0.23–5.73, *p* = 0.862)	-
	3	72 (94.7)	4 (5.3)	1.78 (0.34–13.13, *p* = 0.515)	-
	4	7 (87.5)	1 (12.5)	4.57 (0.20–54.16, *p* = 0.238)	-
Primary	Yes	354 (97.0)	11 (3.0)	-	-
	Recurrent	203 (96.7)	7 (3.3)	1.11 (0.40–2.86, *p* = 0.832)	-
Montreal L	1	236 (98.7)	3 (1.3)	-	-
	2	23 (92.0)	2 (8.0)	6.84 (0.87–43.34, *p* = 0.041)	5.74 (0.70–38.02, *p* = 0.070)
	3	298 (95.8)	13 (4.2)	3.43 (1.09–15.09, *p* = 0.056)	3.32 (1.04–14.75, *p* = 0.066)
Montreal B	1	20 (95.2)	1 (4.8)	-	-
	2	366 (97.6)	9 (2.4)	0.49 (0.09–9.29, *p* = 0.511)	-
	3	171 (95.5)	8 (4.5)	0.94 (0.16–17.81, *p* = 0.951)	-
Previous surgery	No	272 (96.8)	9 (3.2)	-	-
	Yes	284 (96.9)	9 (3.1)	0.96 (0.37–2.49, *p* = 0.928)	-
Timing of surgery	Elective	471 (96.5)	17 (3.5)	-	-
	Urgent	77 (98.7)	1 (1.3)	0.36 (0.02–1.79, *p* = 0.324)	-
	Emergency	8 (100.0)	0 (0.0)	0.00 (NA–NA, *p* = 0.992)	-
Associated surgery	No	424 (97.7)	10 (2.3)	-	-
	Yes	133 (94.3)	8 (5.7)	2.55 (0.96–6.60, *p* = 0.053)	2.81 (1.02–7.64, *p* = 0.040)
Preoperative drainage	No	525 (97.4)	14 (2.6)	-	-
	Yes	32 (88.9)	4 (11.1)	4.69 (1.27–13.96, *p* = 0.009)	5.15 (1.33–16.56, *p* = 0.009)
Preoperative TPN	No	490 (97.0)	15 (3.0)	-	-
	Yes	66 (97.1)	2 (2.9)	0.99 (0.15–3.61, *p* = 0.989)	-
Preoperative therapy with steroids	No	363 (96.0)	15 (4.0)	-	-
	Yes	194 (98.5)	3 (1.5)	0.37 (0.09–1.15, *p* = 0.124)	-
Preoperative therapy with biologics	No	467 (96.3)	18 (3.7)	-	-
	Yes	89 (100.0)	0 (0.0)	0.00 (NA–NA, *p* = 0.989)	-
Preoperative therapy with azathioprine	No	490 (97.0)	15 (3.0)	-	-
	Yes	67 (95.7)	3 (4.3)	1.46 (0.33–4.58, *p* = 0.556)	-

### LOS, readmissions, and reoperations

The median LOS was 7 days (range 3–95) and factors associated with LOS are reported in Table 5. The reoperation rate and readmission rate in the patients who underwent preoperative abscess drainage were 19.4% and 13.9%, respectively.

**Table 5:** Patients’ characteristics and length of stay

		LOS^1^	Coefficient (univariable)	Coefficient (multivariable)
Gender	M	9.3 (8.2)	-	-
	F	8.5 (6.4)	− 0.81 (− 2.08 to 0.45, *p* = 0.2063)	-
BMI		9.0 (7.5)	0.04 (− 0.11 to 0.19, *p* = 0.6079)	-
ASA	1	6.9 (3.8)	-	-
	2	8.6 (6.5)	1.65 (− 0.21 to 3.52, *p* = 0.0817)	0.73 (− 1.08 to 2.53, *p* = 0.4285)
	3	10.6 (7.5)	3.69 (1.33 to 6.06, *p* = 0.0023)	1.23 (− 1.16 to 3.63, *p* = 0.3128)
	4	29.5 (29.9)	22.56 (17.30 to 27.82, *p* < 0.0001)	19.71 (14.26 to 25.16, *p* < 0.0001)
Primary	Yes	8.2 (6.8)	-	-
	Recurrent	10.2 (8.6)	2.03 (0.76 to 3.31, *p* = 0.0018)	1.00 (− 0.22 to 2.22, *p* = 0.1093)
Montreal L	1	8.2 (5.4)	-	-
	2	8.9 (5.9)	0.75 (− 2.36 to 3.86, *p* = 0.6346)	-
	3	9.6 (8.9)	1.40 (0.13 to 2.67, *p* = 0.0313)	-
Montreal B	1	9.2 (5.6)	-	-
	2	8.1 (6.9)	− 1.12 (− 4.41 to 2.16, *p* = 0.5028)	-
	3	10.8 (8.7)	1.60 (− 1.78 to 4.98, *p* = 0.3535)	-
Previous surgery	No	8.5 (7.4)	-	-
	Yes	9.4 (7.7)	0.94 (− 0.30 to 2.18, *p* = 0.1365)	-
Timing of surgery	Elective	8.3 (6.9)	-	-
	Urgent	12.2 (9.1)	3.84 (2.06 to 5.61, *p* < 0.0001)	2.79 (1.04 to 4.55, *p* = 0.0018)
	Emergency	16.2 (15.5)	7.92 (2.74 to 13.10, *p* = 0.0028)	1.22 (− 4.89 to 7.33, *p* = 0.6952)
Associated surgery	No	8.7 (7.0)	-	-
	Yes	9.7 (9.1)	1.04 (− 0.39 to 2.48, p = 0.1537)	-
Preoperative drainage	No	8.7 (7.4)	-	-
	Yes	12.8 (8.8)	4.05 (1.51 to 6.58, *p* = 0.0018)	2.36 (− 0.03 to 4.75, *p* = 0.0527)
Preoperative total parenteral nutrition	No	8.4 (6.8)	-	-
	Yes	12.9 (9.9)	4.55 (2.72 to 6.37, *p* < 0.0001)	1.97 (0.11 to 3.82, *p* = 0.0375)
Preoperative therapy with steroids	No	8.9 (7.0)	-	-
	Yes	9.1 (8.6)	0.16 (− 1.14 to 1.46, *p* = 0.8110)	-
Preoperative therapy with biologics	No	8.9 (7.7)	-	-
	Yes	9.2 (6.6)	0.28 (− 1.43 to 2.00, *p* = 0.7441)	-
Preoperative therapy with azathioprine	No	9.1 (7.9)	-	-
	Yes	7.9 (4.0)	− 1.22 (− 3.11 to 0.67, *p* = 0.2056)	-

^1^Values are reported as mean (standard deviation)

Longer LOS, higher readmissions, and reoperation rates were reported in patients who underwent preoperative abscess drainage.

## Discussion

Patients with CD who require preoperative radiological guided drainage of intra-abdominal abscesses are at increased risk of postoperative morbidity and septic complications following ileocaecal or re-do ileocolic resection. The reason for this reported higher risk of short-term morbidity in this group of patients compared to patients with a fibro stenotic phenotype of CD is likely multifactorial. Penetrating CD poses additional challenges to the surgeons who might need to perform additional resections or repairs on the “target” organs of the fistulating disease. Secondly, the previous admission and treatment for sepsis suggest a more deconditioned patient, likely more prone to postoperative complications, in view of possible malnutrition or extensive inflammatory disease. Not surprisingly, the group of patients requiring abscess drainage prior to surgery demonstrated a higher rate of recurrent disease, penetrating disease, and need for preoperative total parenteral nutrition (TPN), implying a selection of patients with several risk factors for postoperative complications in this group.

Incomplete percutaneous drainage and non-resolution of the intra-abdominal sepsis can affect not only the risk of postoperative abscess recurrence, but also increased postoperative morbidity, and we hope our results could generate discussion on the need for repeated cross-sectional imaging post percutaneous drainage, even if our results did not provide information on the exact dimension of the abscess prior to drainage.

Patients with CD require a multidisciplinary approach [6] for an essential close and structured integration of medical and surgical management to identify the right time for surgery with the aim to prevent emergency surgery, postoperative complications, and recurrence. It is a quality requirement that patients having surgery for IBD have it undertaken by a colorectal surgeon who is a core member of the IBD multidisciplinary team [7] auditing stoma rate, complications, re-interventions, and mortality [8].

The management of intraabdominal or pelvic abscesses usually involves a combination of medical therapy with either percutaneous or surgical drainage. Medical therapy with antibiotics against enteric flora should be initiated and continued after drainage, with the duration dictated by the completeness of the drainage and the subsequent clinical response [9]. When CD patients present with an intra-abdominal abscess resulting from a contained perforation, although resection can theoretically be done as 1 stage with primary anastomosis, this is often not possible in the emergency setting, in view of the high risk of postoperative complications such as anastomotic leak, wound infection, and fistulisation. Complications are also increased when malnutrition, active disease, and/or sepsis coexist [10]. Percutaneous drainage is therefore particularly appealing as a "bridge" to elective surgical intervention allowing for stabilization and optimization with improved outcomes. Retrospective data have shown that primary percutaneous drainage is associated with significantly fewer complications, higher likelihood of successful primary anastomosis, and shorter length of stay [11, 12]. Nevertheless, our study reported a 44.4% 30-day morbidity in patients with CD undergoing surgical resection within 6 weeks of percutaneous drainage of an intra-abdominal abscess, with an 11% anastomotic leak rate and a 19% reoperation rate.

Our study did not evaluate the time gap between drainage and surgery, and no details were collected on abscess’ size. According to Feagins et al. [9], non-drainable abscesses smaller than 3 cm in size with no evidence of fistula and no steroid therapy are likely to respond to antibiotic therapy alone, despite high recurrence rates. Another limitation of our study is the retrospective design and self-reporting nature of the data collection.

Percutaneous drainage can prevent the need for surgery in up to 30% of selected CD patients with the use of anti-tumor necrosis factor agents being associated with the higher success of conservative management [13], once sepsis had resolved [14], but our study cannot provide guidance on the outcomes of patient who did not require surgery following drainage, as these have not been captured. A recent multicentre study including 335 CD patients with percutaneous drainage followed by surgery [15] reported a complication rate of 32.2%, with residual abscess, low serum albumin concentration [16], and an interval of less than 2 weeks between drainage and surgery, being associated with higher risk of complications. It is important to note that a 25% abscess persistence rate has been reported at surgery after the resolution of the acute episode with percutaneous drainage [17], and this should reiterate the need for re-imaging in patients where planned surgical treatment is avoided.

## Conclusions

Percutaneous drainage is a valid treatment option as a bridge to surgery in patients with CD complicated by intra-abdominal abscess. Despite percutaneous drainage, surgical treatment maintains a high risk of morbidity and septic complications.

## Supplementary Information

Below is the link to the electronic supplementary material.

Supplementary file1 (DOCX 17 KB)

Supplementary file2 (DOCX 13 KB)
